# Pose Self-Calibration of Stereo Vision Systems for Autonomous Vehicle Applications

**DOI:** 10.3390/s16091492

**Published:** 2016-09-14

**Authors:** Basam Musleh, David Martín, José María Armingol, Arturo de la Escalera

**Affiliations:** Intelligent Systems Laboratory, Universidad Carlos III de Madrid/Avda de la Universidad 30, Leganés, Madrid 28911, Spain; dmgomez@ing.uc3m.es (D.M.); armingol@ing.uc3m.es (J.M.A.); escalera@ing.uc3m.es (A.E.)

**Keywords:** camera pose estimation, stereo vision, advanced driver assistance systems, intelligent vehicles

## Abstract

Nowadays, intelligent systems applied to vehicles have grown very rapidly; their goal is not only the improvement of safety, but also making autonomous driving possible. Many of these intelligent systems are based on making use of computer vision in order to know the environment and act accordingly. It is of great importance to be able to estimate the pose of the vision system because the measurement matching between the perception system (pixels) and the vehicle environment (meters) depends on the relative position between the perception system and the environment. A new method of camera pose estimation for stereo systems is presented in this paper, whose main contribution regarding the state of the art on the subject is the estimation of the pitch angle without being affected by the roll angle. The validation of the self-calibration method is accomplished by comparing it with relevant methods of camera pose estimation, where a synthetic sequence is used in order to measure the continuous error with a ground truth. This validation is enriched by the experimental results of the method in real traffic environments.

## 1. Introduction

Nowadays, according to WHO (World Health Organization), traffic accidents are one of the main causes of death in the world, ranking ninth. Quantitatively, traffic accidents accounted for nearly 1.3 million deaths in 2012 in the world [1]. In the most current available data (2014), in the European Union, the number of deaths from this cause was approximately 25,700 people [2]. For this reason, there is a continuous social demand for improving road safety due to the high socio-economic cost of road accidents, being one of the major ones responsible for the deep development, that has taken place, in the implementation of security systems in everything related to the automotive industry, either by manufacturers, authorities or researchers in this field. Initially, the systems belonging to passive safety experienced very rapid growth by manufacturers, an example of such being the safety belt or airbag, while active systems have undergone a more late development, due to the complexity and the need for reliability close to 100%, where the ABS (In German, Antiblockiersystem) was one of the first systems of this type introduced by manufacturers. Current examples of active safety systems based on computer vision are, among others: the blind spot monitor, traffic sign recognition or driver drowsiness detection.

Active safety systems include ADASs (Advance Driver Assistance Systems), which are mainly based on the use of sophisticated sensory systems, whose aim is to extract information from the vehicle environment and react accordingly. These systems typically perform monitoring tasks of the driving behavior until a dangerous situation occurs; then, the system generates an alert to the driver to warn of such a dangerous situation. Traditionally, these systems do not activate the vehicle components, such as brakes or steering, but in recent vehicles, this trend is changing, and it is possible to find basic solutions commercially implemented. This requires a high degree of effectiveness of the algorithms, from the viewpoint of reliability and response time, an extreme case being when the algorithms are applied to autonomous vehicles [3,4,5]. The future implementation of so-called intelligent or autonomous vehicles will allow a significant improvement in road safety, due to the elimination of one of the main causes of traffic accidents, that is the human factor [6], in addition to improving traffic management. As an example, the City of Madrid (Spain) estimated in 2010 that the number of lost hours a day in traffic jams amounted to 329,000 assuming a cost of 3.4 million € per day.

**Figure 1:**
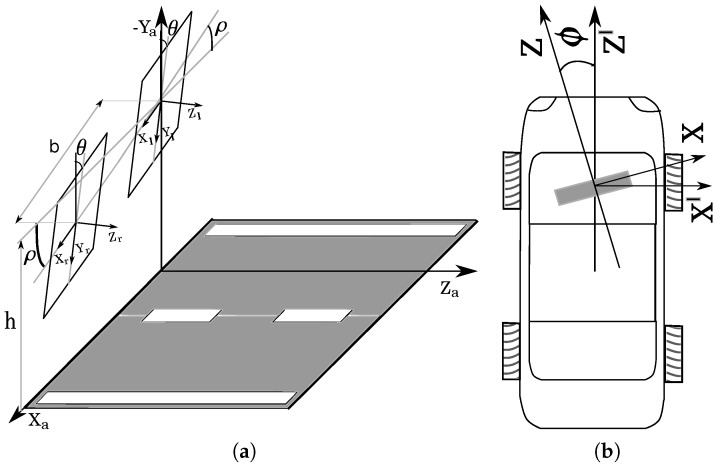
(**a**) Schema of the configuration of the stereo rig in relation to the ground; (**b**) schema of the yaw deviation (*ϕ*).

One of the main problems of ADASs is correlating the sensor measurements with the real world, i.e., the environment surrounding the vehicle. Computer vision systems are based on cameras, and therefore, the measurements are made in pixels (camera coordinate system), where pixels must be converted to meters (world coordinate system). This topic is the main contribution of this paper, since the self-calibration of the extrinsic parameters of the stereo system has a major impact on the accuracy of the world coordinates. Cameras or vision systems can be placed in different positions of the vehicle, depending on the task performed by the ADAS [7]. The measurement matching between the perception system (pixels) and the vehicle environment (meters) depends on the relative position between the perception system and the environment. This relative position is normally expressed regarding the road ahead of the vehicle (see Figure 1a), where the relative position problem, analysis and solution are named in the literature as ‘camera pose estimation’. Thus, it is necessary to know the relative position of the perception system with respect to the road in real time to obtain precise measurements of the vehicle environment, because the nominal values change due to irregularities that appear in the roadway. The in-vehicle systems where it is fundamental to perform accurate measurements are the following: emergency stop systems to avoid frontal collisions or automatic manoeuvres to generate non-collision trajectories. Moreover, most of these in-vehicle systems are being implemented in autonomous navigation.

The structure of the paper is as follows: the state of the art is detailed in the next section (Section 2), where there are three main groups of methods for estimating the camera pose estimation: the first one is the calibration patterns; the second is the road markings as a source of information; and finally, the geometry of the road ahead of the vehicle. The developed algorithm in this paper (explained in Section 3) belongs to the third group of methods, being an evolution of the work presented in [8]. The performance of the presented method is assessed in Section 4, where the main contribution of our algorithm regarding the state of the art on the subject is the estimation of the pitch angle (*θ*) without being affected by the roll angle (*ρ*). To illustrate this advantage, the presented algorithm is compared with a work where the pitch angle was affected by the roll angle [9] (see Section 3.3.1). Moreover, a synthetic sequence has been used to accomplish the explained comparison [10], where pitch and roll angle changes appear in the whole sequence. Finally, the presented algorithm is compared with a camera pose estimation algorithm of the bibliography, which is also based on the use of the geometry of the roadway in front of the vehicle [11].

## 2. State of the Art

There are different techniques or methods that establish the relative position of the vision system with respect to the surrounding environment, either monocular [12,13] or stereo, where, in ITS (Intelligent Transportation Systems) applications, the position of the vision system is usually determined regarding the roadway or the road in front of the vehicle. In these techniques, the relative position of the vision system is determined by knowing the orientation angles (yaw (*ϕ*), pitch (*θ*) and roll (*ρ*)) and the height (*h*) of the camera with respect to the road (see Figure 1a,b). These three angles and the height are known as the extrinsic parameters of the vision system. The values of these extrinsic parameters change considerably while the vehicle is moving; this is mainly due to changes in the vehicle speed (i.e., acceleration or braking) or when irregularities appear in the roadway, like potholes and speed bumps [14].

The methods for self-calibration of the extrinsic parameters can be divided into three groups based on the type of information that is utilized in the process:

- Calibration patterns: In this first group of calibration methods, patterns are taking into account for determining the extrinsic parameters of the vision system. These methods are based on minimizing the projection error of a number of known points located around the vehicle, which are joined in order to create the pattern. These patterns may be located on the ground [15,16] or painted on the hood of the vehicle [17].
- Road marks: Secondly, the calibration process is performed by means of road marks [18], such as lines [19,20,21] or dashed lines on the roadway [22], it being possible to use the parking lines as the calibration pattern [23]. These methods allow the calibration process, where it is possible to recalculate the extrinsic parameters at different times and positions. The inconvenience of the road marks is related to the impossibility to be constantly detected, for example in urban environments, where road marks can be found in poor conservation or occluded by other elements, such as parked vehicles, but above all, the fact that there are few road marks within cities.
- The geometry of the road in front of the vehicle: The last group of methods is based on estimating the geometry of the roadway in front of the vehicle, which can be accomplished mainly in two different ways. Firstly, the three-dimensional information of the vehicle environment contained in the disparity map allows one to determine the position of the ground in front of the vehicle by means of different kinds of projections. While a second technique is based on the sampling of 3D points and subsequent adjustment to a plane [24], where both techniques can be used in outdoor applications [25,26]. Such methods allow one to find out the extrinsic parameters, avoiding the need for the calibration pattern or the road marks. Moreover, this allows recalculating the relative position of the vision system in real time while the vehicle is moving and adapting to changing parameters, as discussed above, such as vehicle load, acceleration or irregularities of the roadway.

There are several techniques designed for obtaining the extrinsic parameters of the disparity map, which the work presented by [27] highlights for determining the pitch angle (*θ*) and height (*h*), and the subsequent improved technique for obtaining the information of the roll angle (*ρ*) [28]. These works are based on the use of the projection of the disparity map on the vertical axis of the image (v), which is known as v-disparity, and obtaining the road profile as a straight line in the v-disparity by Hough transform. The second reference work for obtaining information on the extrinsic parameters (pitch (*θ*), roll (*ρ*) and height (*h*)) from the disparity map is presented in [11], and the following extension to non-planar grounds [29], which is based on the construction of the so-called virtual disparity map, which corresponds to the resulting disparity map if the stereo system could be positioned at the height of the road, that is height h=0. Once the virtual disparity map is constructed, the algorithm is similar to that presented by [27] for estimating the pitch angle (*θ*) and height (*h*), being different in the case of the roll angle (*ρ*), but determining the variations that occur in the values of the extrinsic parameters between consecutive images.

Finally, referring to the estimation of the extrinsic parameters by using points in world coordinates (3D) and subsequent adjustment to a plane, some authors use a previous selection of points in the plane YOZ, formed by the axes (Y,Z), to reduce the number of outliers in the final adjustment of the plane, as well as the computational load of the algorithm [14,30]. Other works, as presented in [31], estimate directly the roadway profile in the plane YOZ, simplifying the problem to an adjustment in two dimensions; then, the method is similar to the v-disparity, but replacing the disparity by the depth (*Z*). A comparison between both methods can be found in [32]. On the other hand, some works presented obtain information relative to the plane of the roadway ahead of the vehicle by calculating the homography between the images of the stereo pair [33]. The necessary condition for the calculation of such homography is using only the area, in both images, where the roadway appears. The roadway can be detected by means of a segmentation process based on color, as in [34,35], or using only a region of interest near the vehicle where it is possible to assume that almost the whole region of interest corresponds to the roadway [36].

## 3. Extrinsic Parameter Self-Calibration

The self-calibration algorithm is divided into two different stages. In the first step, the yaw deviation (*ϕ*) between the stereo system and the vehicle movement is estimated, and it is assumed consistent over time. This deviation is calibrated while the vehicle is performing a straight-line motion (Figure 1b). Secondly, the remaining extrinsic parameters, i.e., the height (*h*), pitch (*θ*) and roll (*ρ*), are calculated. This second stage is performed on every image captured by the stereo system in order to detect the variations in the value of these extrinsic parameters.

### 3.1. System Configuration

The computer vision system used in this research is a stereo system composed of two cameras whose image planes are ideally coplanar in order to make it possible that the epipolar lines can be parallel. However, a rectification process is necessary [37] because the coplanar assumption is not correct in real applications. A schema of the configuration of the stereo rig and the road (ground) is shown in Figure 1a. It is important to explain that the yaw deviation (*ϕ*) shall be assumed to be zero. In the case of existing yaw deviation (*ϕ*) (see Figure 1b), this is estimated in the first stage of the self-calibration algorithm, as is described in Section 3.2.

As shown in Figure 1a, the stereo system is situated at a height *h* above the ground, and the stereo rig is rotated an angle *θ* (pitch) around the axis *X* and an angle *ρ* (roll) around the axis Z, which correspond to the angles between the stereo rig and the ground. The baseline between the cameras of the stereo system is (*b*). Making use of the system scheme (see Figure 1a), it is possible to relate the homogeneous coordinates of the world of a point P=(X,Y,Z,1) to its projection coordinates $p=(u_{i}S,vS,S,1)$ in both image planes (both cameras) by means of Equation (1), where *M* corresponds to homogeneous matrices of transformation (2); in particular, $M_{Tk}$ are translation matrices along the axis *k* and $M_{Rk}$ are rotation matrices around the axis *k*; whereas $M_{P}$ is the camera projection matrix, which is defined by the focal length in pixels (*α*) and the coordinates of the principal point ($u_{0},v_{0}$). In the expression (1), the coordinates of the projection on each camera are defined by *j* in such a way that j=r for the right camera and j=l for the left one. A developed expression (3) for the relationship between world coordinates and image coordinates is obtained as a result of the multiplication of the matrices in Equation (1).

(1) $$\left[\begin{array}{c} u_{j}S\\ vS\\ S\\ 1 \end{array}\right]=M_{P}\left(\alpha ,u_{0},v_{0}\right)\cdot M_{Tx}\left(\varepsilon_{j}b\right)\cdot M_{Rx}\left(\theta \right)\cdot M_{Rz}\left(\rho \right)\cdot M_{Ty}\left(h\right)\left[\begin{array}{c} X\\ Y\\ Z\\ 1 \end{array}\right]$$

(2) $$\begin{array}{cc} M_{P}\left(\alpha ,u_{0},v_{0}\right)=\left[\begin{array}{cccc} \alpha & 0 & u_{0} & 0\\ 0 & \alpha & v_{0} & 0\\ 0 & 0 & 1 & 0\\ 0 & 0 & 0 & 1 \end{array}\right] & \;M_{Tx}\left(\varepsilon_{j}b\right)=\left[\begin{array}{cccc} 1 & 0 & 0 & -\varepsilon_{j}b\\ 0 & 1 & 0 & 0\\ 0 & 0 & 1 & 0\\ 0 & 0 & 0 & 1 \end{array}\right]\\ M_{Rx}\left(\theta \right)=\left[\begin{array}{cccc} 1 & 0 & 0 & 0\\ 0 & \cos\theta & -\sin\theta & 0\\ 0 & \sin\theta & \cos\theta & 0\\ 0 & 0 & 0 & 1 \end{array}\right] & \;M_{Rz}\left(\rho \right)=\left[\begin{array}{cccc} \cos\rho & -\sin\rho & 0 & 0\\ \sin\rho & \cos\rho & 0 & 0\\ 0 & 0 & 1 & 0\\ 0 & 0 & 0 & 1 \end{array}\right]\\ M_{Ty}\left(h\right)=\left[\begin{array}{cccc} 1 & 0 & 0 & 0\\ 0 & 1 & 0 & h\\ 0 & 0 & 1 & 0\\ 0 & 0 & 0 & 1 \end{array}\right] & \end{array}$$

(3) $$\begin{array}{cc} u_{j}S= & X\left(\alpha \cos\rho +u_{0}\sin\rho \sin\theta \right)-\left(Y+h\right)\left(\alpha \sin\rho -u_{0}\cos\rho \sin\theta \right)+\\ & \;\;\;+Z\left(u_{0}\cos\theta \right)-\varepsilon_{j}\alpha b\\ vS= & \left[\alpha \cos\theta +v_{0}\sin\theta \right]\left(\left(Y+h\right)\cos\rho +X\sin\rho \right)-Z\left(\alpha \sin\theta -v_{0}\cos\theta \right)\\ S= & Z\cos\theta +(Y+h)\cos\rho \sin\theta +X\sin\rho \sin\theta \end{array}$$

Stereo systems have a great advantage over monocular systems because it is possible to estimate the depth (*Z*) for every point of the world that appears projected on both images. The depth (*Z*) where every point (*P*) is located in the world is a function of its disparity value (Δ), which corresponds to the difference between the horizontal coordinate of the projection of the point (*P*) on both images (4). The value of both $u_{l}S$ and $u_{r}S$ may be calculated by Equation (3), obtaining, thus, a new expression for the disparity (Δ) (5) whose value is dependent exclusively on the world coordinates of the point P=(X,Y,Z) and the extrinsic parameters (ρ,θ,h) and intrinsic parameters (α,b) of the stereo system.

(4) $$\Delta =u_{l}-u_{r}=\frac{u_{l}S-u_{r}S}{S}$$

(5) $$\Delta =\frac{\alpha \cdot b}{S}=\frac{\alpha \cdot b}{Z\cos\theta +(Y+h)\cos\rho \sin\theta +X\sin\rho \sin\theta }$$

Relating the world coordinates of a point (*P*) to its image coordinates (1) is as interesting as knowing the inverse relationship. In other words, by knowing the image coordinates of the projection of a point on one of the image planes $\left(u_{j},v\right)$ (left or right camera), the value of the world coordinates of this point (X,Y,Z) can be calculated. It is possible to express this inverse relationship as Equation (6), where the value of the world coordinates is dependent on *S*, which, in turn, is a function of them (see Equation (3)), which invalidates the objective of relating the coordinates of the image with the coordinates of the world. In order to overcome this difficulty, it is possible to make use of Equation (5) of the disparity (Δ) and to express $S=\frac{\alpha \cdot b}{\Delta }$. In this way, the world coordinates are not dependent on *S*, making it possible to calculate the coordinates of the world (X,Y,Z) by knowing the values of the image coordinates of the projection $\left(u_{j},v\right)$, the disparity (Δ) and the intrinsic parameters (α,b) and extrinsic parameters (ρ,θ,h) of the stereo system. Equations (7) and (8) express this new relationship between each world coordinate (X,Y,Z) and the coordinates of the image $\left(u_{j},v\right)$.

(6) $$\left[\begin{array}{c} X\\ Y\\ Z\\ 1 \end{array}\right]=M_{Ty}^{-1}\left(h\right)\cdot M_{Rz}^{-1}\left(\rho \right)\cdot M_{Rx}^{-1}\left(\theta \right)\cdot M_{Tx}^{-1}\left(\varepsilon_{j}b\right)\cdot M_{P}^{-1}\left(\alpha ,u_{0},v_{0}\right)\cdot \left[\begin{array}{c} u_{j}S\\ vS\\ S\\ 1 \end{array}\right]$$

(7) $$\left[\begin{array}{c} X\\ Y\\ Z\\ 1 \end{array}\right]=M_{Ty}^{-1}\left(h\right)\cdot M_{Rz}^{-1}\left(\rho \right)\cdot M_{Rx}^{-1}\left(\theta \right)\cdot M_{Tx}^{-1}\left(\varepsilon_{j}b\right)\cdot M_{P}^{-1}\left(\alpha ,u_{0},v_{0}\right)\cdot \left[\begin{array}{c} u\frac{\alpha b}{\Delta }\\ v\frac{\alpha b}{\Delta }\\ \frac{\alpha b}{\Delta }\\ 1 \end{array}\right]$$

(8) $$\begin{array}{c} X=\varepsilon_{j}b\cos\rho +\left(b\cos\theta \sin\rho \left(v-v_{0}\right)+b\cos\rho \left(u-u_{0}\right)+\alpha b\sin\rho \sin\theta \right)/\Delta \\ Y=-\varepsilon_{j}b\sin\rho -h+\left(b\cos\rho \cos\theta \left(v-v_{0}\right)-b\sin\rho \left(u-u_{0}\right)+\alpha b\cos\rho \sin\theta \right)/\Delta \\ Z=\left(\alpha b\cos\theta -b\sin\theta (v-v_{0})\right)/\Delta \end{array}$$

At this point, it is possible to determine an expression (9) that is fulfilled for all points of the ground or road in front of the vehicle. A flat road geometry is assumed in such a way that Y=0 for every point of the road (see Equation (8)). Equation (9) determines the relationship fulfilled by the image coordinates (u,v) (left image $\varepsilon_{l}=0$) of points belonging to the road according to the values of disparity (Δ) and extrinsic and intrinsic parameters of the stereo system.

(9) $$\left(v-v_{0}\right)=\frac{\tan\rho }{\cos\theta }\cdot \left(u-u_{0}\right)-\alpha \tan\theta +\left(\frac{h}{b\cos\rho \cos\theta }\right)\Delta$$

### 3.2. Yaw Calibration (ϕ)

The calibration of the yaw angle (see Figure 1b) is based on detecting the vanishing point in two consecutive images, while the vehicle performs a straight line movement. Ideally, if there is no deviation of the yaw angle, the horizontal coordinate of the vanishing point ($u_{vp}$) must be equal to the horizontal coordinate of the optical center ($u_{0}$). Thus, if there is a deviation, this can be estimated by Equation (10). The determination of the vanishing point is calculated by matching points of interest between two consecutive images. Then, assuming that the coordinates of the image of a point *n* in the first image are $\left(u_{n_{1}},v_{n_{1}}\right)$ and the second are $\left(u_{n_{2}},v_{n_{2}}\right)$, the straight line $r_{n}$ that passes through the two points can be calculated using the following expression (11). The coordinates of the vanishing point are the intersection point of all straight lines.

(10) $$\phi =\arctan\left(\frac{u_{vp}-u_{0}}{\alpha }\right)$$

(11) $$v=\frac{v_{n_{2}}-v_{n_{1}}}{u_{n_{2}}-u_{n_{1}}}\cdot u+v_{n_{2}}\left(u_{n_{2}}+u_{n_{1}}\right)$$

**Figure 2:**
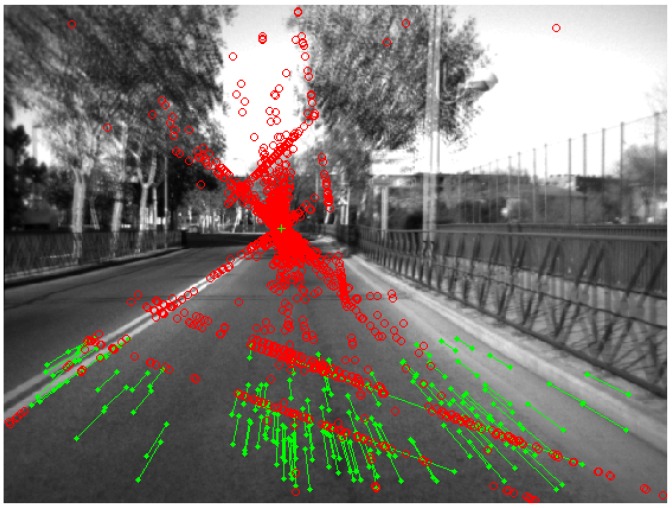
Calibration of the deviation of the yaw angle in an urban environment: superposition of two consecutive images, where the consecutive positions of each point of interest are connected by green lines, while the intersections of the straight lines appear as red circles.

The calibration process is carried out in an uncontrolled environment, such as urban environments, where dynamic elements can appear in front of the vehicle, such as moving vehicles or pedestrians, among others. This environment complicates the determination of the vanishing point by the process described previously. Then, only points of interest that belong to the ground or roadway are used in order to mitigate the effect of the dynamic elements to determine the vanishing point. These points are obtained from the free map, that is a disparity map where obstacles have been removed therefrom, and therefore, the free map contains mainly depth information of the roadway (for more information on obtaining the free map, see [9]). In addition to this source of errors, there are others, such as the vibration of the vehicle or mismatches of points of interest between two consecutive images. For this reason, the vanishing point is selected by means of all possible solutions, which are obtained from the intersection of the complete set of pairs of lines. The algorithm to select one point from all sets of points is RANSAC [38]. This process is repeated continuously using several images, and the final vanishing point is selected again using the RANSAC algorithm. An example of the implementation of the yaw angle calibration is presented in Figure 2, where the position of each point of interest in the two consecutive images is joined by green lines, so the corresponding vanishing point is obtained by the intersection of all straight lines.

### 3.3. Self-Calibration of the Height (h) and the Pitch (θ) and Roll (ρ) Angles

Once the deviation of the yaw (*ϕ*) has been estimated (see Figure 1b), the configuration of the stereo rig in relation to the road is shown in Figure 1a, and thus, all of the mathematical formulation developed in the preceding Section 3.1 can be applied. The values of the extrinsic parameters of the stereo system contain the information regarding the camera pose in relation to the road. For this reason, it is necessary to detect the road at all times in order to update the possible variations in the extrinsic parameters. As explained earlier, every pixel of the disparity map and, thus, of the visible image (left image $\varepsilon_{l}=0$) that belong to the road fulfil Equation (9). This expression represents the relationship between image coordinates, which fits to the equation of a straight line (12) for the different values of disparity (Δ). Equations (13) and (14) correspond to the slope (*c*) and to the y-intercept (*d*) of the straight line (12), respectively.

(12) $$\left(v-v_{0}\right)=c\cdot \left(u-u_{0}\right)+d$$

(13) $$c=\frac{\tan\rho }{\cos\theta }$$

(14) $$d=-\alpha \tan\theta +\left(\frac{h}{b\cos\rho \cos\theta }\right)\Delta$$

Two different situations may arise in the self-calibration of the extrinsic parameters of the stereo system. Firstly, there are situations where the roll (*ρ*) has little impact (see Section 3.3.1), so it is possible to assume that (ρ=0) in the mathematical formulation previously developed, whereas there are other situations where the previous simplification (to assume that (ρ=0)) is not possible and the method in order to estimate the values of the extrinsic parameters of the stereo system shall be different (see Section 3.3.2).

#### 3.3.1. Self-Calibration for Negligible Values of the Roll Angle (Method 1)

The roll values are normally low (±5°) in urban environments, except the cases associated with abnormal inclinations of the roadway or closed curves. Thus, if a null value of the roll angle (ρ=0) is considered, then it is possible to simplify the expression (9), obtaining a new expression (15) for pixels belonging to the roadway, which, in this case, relate the vertical coordinate of the image with the disparity (Δ). This simplified expression also corresponds to the equation of a straight line, and in this case, the equation is $v=C_{r}\Delta +v_{\Delta 0}$, where $C_{r}$ is the slope and $v_{\Delta 0}$ is the value of (*v*) if the disparity is Δ=0. This straight line is known as the road profile, and therefore, the line parameters ($C_{r},v_{\Delta 0}$) can be extracted from v-disparity, as is detailed in the work [27]. Once this line is detected in the v-disparity, the value of the parameters are obtained ($C_{r},v_{\Delta 0}$), and finally, the value of the searched extrinsic parameters can be estimated: the pitch angle (16) and the height of the stereo system to the roadway (17).

(15) $$v=\frac{h}{b\cos\theta }\cdot \Delta +v_{0}-\alpha \tan\theta$$

(16) $$\theta =\arctan\left(\frac{v_{0}-v_{\Delta 0}}{\alpha }\right)$$

(17) $$h=C_{r}\cdot b\cdot \cos\left(\theta \right)$$

**Figure 3:**
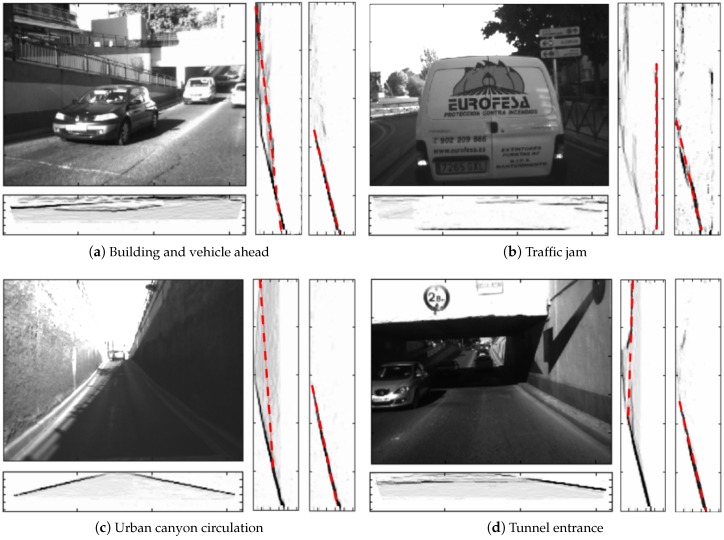
Examples of uv-disparity for different scenarios of interest in urban environments, where the left visible image, on the bottom the corresponding u-disparity, and right, the v-disparity and the v-disparity-free with the detection of the road profile (dashed red line), are shown.

Two typical methods to obtain the road profile in the v-disparity are the application of the Hough transform for straight lines and the adjustment of straight lines by using RANSAC. Both methods obtain, as a result, the most voted for straight line, that is the straight line that contains a greater amount of points in the v-disparity. There are several works, such as [39,40,41], that explain the difficulties caused by the obstacles in determining the road profile, being a complex case when it does not match with the straight line with more points in the v-disparity (see Figure 3). For example, this difficulty arises when one or more large obstacles are in front of the vehicle, which is very common in urban environments. In our previous work [9], a variant was presented to the method presented in [27] to obtain the road profile, where in this case, a different v-disparity called ‘v-disparity-free’ was used, characterized by the fact that the obstacles have been removed therefrom. In order to obtain the v-disparity-free, the free map is utilized, which is a dense disparity map where all pixels belonging to the obstacles have been removed. Thus, the number of times for which the road profile is not the most voted straight line in the v-disparity-free is greatly reduced.

Figure 3 presents several examples of urban scenarios, where the use of the free v-disparity allows the determination of the road profile, which it is impossible to obtain by using only the v-disparity [27]. The first scenario, and one of the most frequent, is when a large obstacle suddenly appears in front of the vehicle, such as vehicles or buildings. Figure 3 shows two different cases where a vehicle appears ahead of the stereo camera. In Figure 3a, a vehicle approaching and a wall behind can be observed, while Figure 3b shows a typical image of a traffic jam. The problem arises because the line of the obstacle is the most important in the v-disparity, due to the obstacle covering a large area of the image. In both cases, the u-disparity is presented below the visible images, where it is possible to distinguish the obstacles clearly. The two v-disparities for each case are also shown: the first (left) is generated by using the disparity map, the obtained road profile being wrong (dashed red line). However, in the second case (right), the v-disparity-free has been generated from the free map, where there are no obstacles, so the road profile is now correct. The following example illustrates a special case: two large walls on either side of the vehicle, as shown in Figure 3c. Both walls appear clearly in the u-disparity as two long oblique lines and, therefore, both walls are easily detectable as obstacles. The problem arises due to the two walls being also in the v-disparity, so the obtained road profile in the v-disparity, which is generated from the disparity map, is wrong. The v-disparity-free is developed in order to solve this problem and so to find the road profile correctly. The fact that circulating under high obstacles, such as semaphores or at the entrance to a tunnel (Figure 3d), is a typical action for the circulation of vehicles, complex positioning cases appear and can be solved by the use of v-disparity-free.

**Figure 4:**
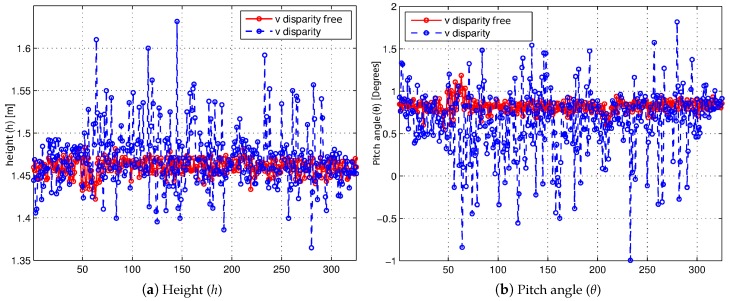
Evolution of estimated extrinsic parameters (*h*,*θ*) along the synthetic sequence [10] using the v-disparity (blue) and the v-disparity-free (red), respectively.

In order to analyze, in a quantitative way, the effectiveness of the use of the v-disparity-free against the use of v-disparity, a synthetic sequence is used in this work [10]. Obstacles such as buildings and vehicles appear along the sequence, and therefore, these obstacles have an impact on the self-calibration process. It is assumed that both the height (*h*) at which the stereo camera is positioned and the pitch angle (*θ*) are constant (the roll angle (*ρ*) is zero) through the 325 images of the synthetic sequence. Figure 4 presents the evolution of the results of the estimation of both the height of the stereo system (Figure 4a) and the pitch angle (Figure 4b), respectively, where both the v-disparity (blue line) and the v-disparity-free (red line) have been used. As can be seen, in both graphs, the estimates resulting from the use of v-disparity-free exhibit less variability in the results, which can be quantified by observing the standard deviations of the results. Using the v-disparity, an average height of 1.47 m with a standard deviation of 0.0360 m has has obtained, while the use of v-disparity-free has obtained an average height of 1.46 m with a standard deviation of 0.0095 m, which represents a reduction of approximately 75%. On the other hand, the reduction in the standard deviations of pitch angle (*θ*) is close to 80% when using the v-disparity-free (σ=0.0725°) instead of using the v-disparity (σ=0.3985°).

**Figure 5:**
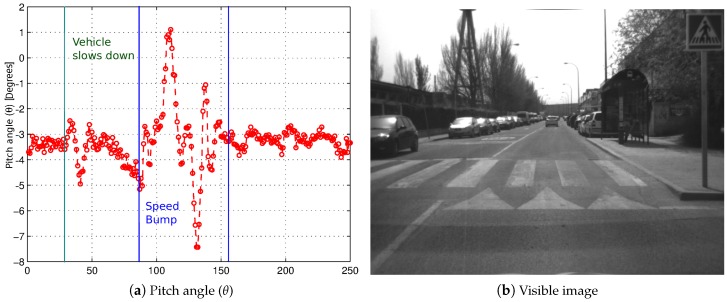
Result of the estimated pitch angle when the vehicle passes through a speed bump.

Once having justified the advantages of using v-disparity-free instead of v-disparity, both for the estimation of the height (*h*) and the pitch angle (*θ*) in the absence of high roll angles, the following is the result of pitch angle (*θ*) estimation in a real case where considerable variations appear: the vehicle is traveling on a speed bump (Figure 5b). As shown in Figure 5a, in this urban scenario, important variations occur in the pitch angle (*θ*) due to changes in the roadway. Moreover, these variations contain a relevant oscillatory component due to the suspension of the vehicle. The variation that occurs in the pitch angle when the vehicle slows down as it approaches the speed bump is also noteworthy.

#### 3.3.2. Self-Calibration for Non-Negligible Values of the Roll Angle (Method 2)

**Figure 6:**
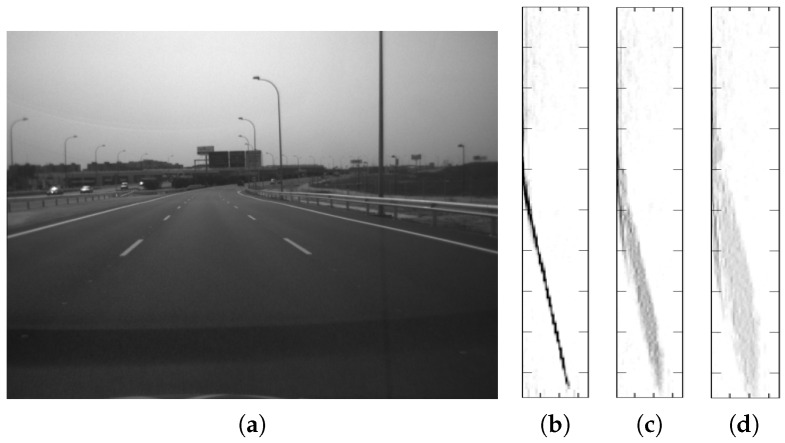
Assessment of the impact that the roll angle (*ρ*) has on the v-disparity and on the road profile. (**a**) Visible image of a highway traffic scenario; (**b**) v-disparity; (**c**) v-disparity after adding a 9° roll angle; (**d**) v-disparity after adding an 18° roll angle.

**Figure 7:**
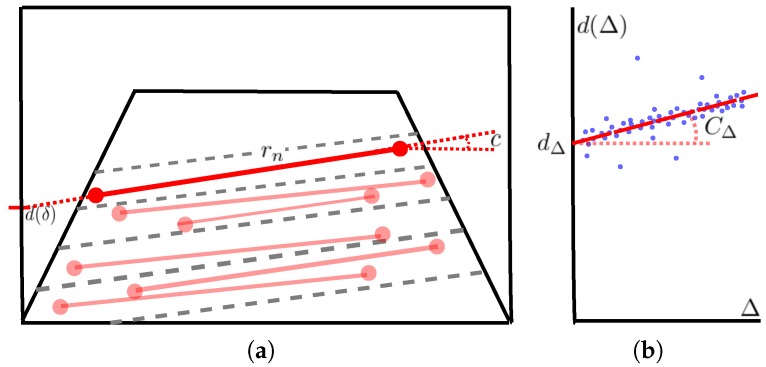
Schema of the different stages of the self-calibration method of the extrinsic parameters (h,θ,ρ) when the roll angle (*ρ*) has non-negligible values. (**a**) Schema of the first stage of the method, which shows the straight lines (lines in red) that join the points (points in red) belonging to the road in the free map for each level of disparity; (**b**) schema of the second stage, which depicts how to fit the linear equation (dashed line in red) resulting from the point cloud data ${\left\{d\left(\delta \right)\right\}}_{n}$ (points in blue).

It is not possible to simplify the expression (9) in order to obtain the equation of the road profile (15) when the roll angle has non-negligible values (see Figure 6), and therefore, it is necessary to provide a reliable method to self-calibrate the extrinsic parameters (h,θ,ρ) as an alternative to the method previously explained in Section 3.3.1. This paper presents a new method of camera pose estimation for stereo systems, whose key feature is the fact that it allows one to estimate the pitch angle (*θ*) for high values of the roll angle (*ρ*) in relation to the ground. The estimation of the pitch angle (*θ*) is based on examining the relationship between the y-intercept (*d*) (14) of the expression (9) for the different values of disparity (Δ). This relationship is a linear equation (see Figure 7b) as described in (18), and thus, if it is possible to calculate ($d_{\Delta }$) and ($C_{\Delta }$), then it will be possible to estimate the values of the extrinsic parameters (h,θ,ρ).

(18) $$d\left(\Delta \right)=C_{\Delta }\cdot \Delta +d_{\Delta }\;\mathrm{where}\;d_{\Delta }=-\alpha \tan\theta \;\mathrm{and}\;C_{\Delta }=\left(\frac{h}{b\cos\rho \cos\theta }\right)$$

The approach implemented with a view toward estimating the coefficients of the linear equation which fits the expression (18) is based on calculating the values of d(Δ) for the different levels of disparity (Δ). To this end, we generate a point cloud obtained by means of the pixels belonging to the free map, due to only the road points fulfilling Equation (9) and, thus, Equation (18). The method for the camera pose estimation follows the procedure outlined in the next steps:

1. Firstly, all pixels of the free map for each possible level of disparity (Δ=δ) are gathered together in pairs of points. A linear equation $r_{n}$ is obtained by using each pair of points. All of these linear equations fulfil the expression (12) (see Figure 7a), and therefore, it is possible to achieve a pair ${\left[c,d\left(\delta \right)\right]}_{n}$ from the slope and the y-intercept of each linear equation $r_{n}$.
2. Once the first stage has been completed for every pixel of the free map, a solution set (${\left\{c\right\}}_{n},{\left\{d\left(\delta \right)\right\}}_{n}$) has been gathered together both for the slope (*c*) and the y-intercept (d(Δ)) of linear Equation (12). The solution set (${\left\{d\left(\delta \right)\right\}}_{n}$), in turn, takes the form of a point cloud, which is possible to fit to a linear equation that fulfills the expression (18), obtaining the values both of ($d_{\Delta }$) and of ($C_{\Delta }$) (see Figure 7b). The value of the pitch angle (*θ*) is estimated directly from ($d_{\Delta }$) by means of Equation (19).
  (19) $$\theta =\arctan\left(-\frac{d_{\Delta }}{\alpha }\right)$$
3. The roll angle (*ρ*) is thereupon estimated by means of the solution set (${\left\{c\right\}}_{n}$) of the slope (*c*) (see Equation (13)), where the optimum solution can be achieved by using RANSAC [38]. It is possible to estimate the roll angle (*ρ*) by using (20) due to the value of the pitch angle (*θ*) being calculated as a result of the second stage. Finally, the remaining extrinsic parameter *h* (height) may be estimated from the value of $C_{\Delta }$ (see Equation (18)) and the pitch (*θ*) and roll (*ρ*) angles by means of Equation (21).
  (20) $$\rho =\arctan\left(c\cdot \cos\theta \right)$$
  (21) $$h=C_{\Delta }\cdot b\cos\rho \cos\theta$$

**Figure 8:**
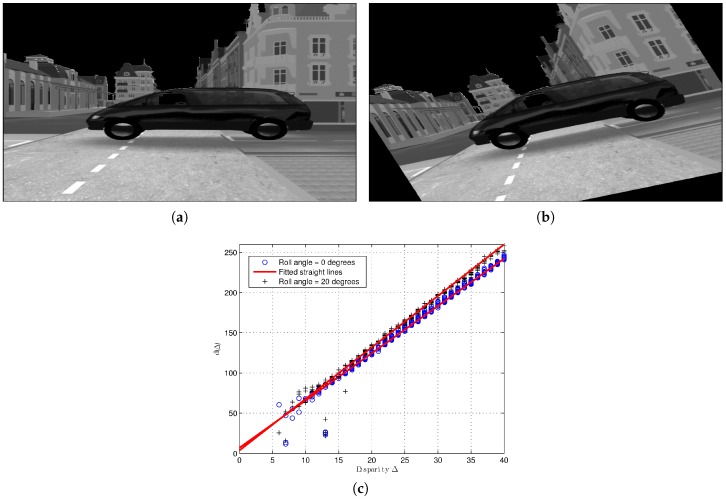
Example of the partial results of the self-calibration Method 2 for two different values of roll angle. (**a**) Visible image of a synthetic traffic scenario [10] in an urban environment with a value of roll angle equal to 0°; (**b**) visible image of a synthetic traffic scenario in an urban environment with a value of roll angle equal to 20°; (**c**) point clouds obtained from each value of roll angle with their respective straight line.

Figure 8 shows two examples of Method 2’s implementation for the same traffic scenario with two different values of roll angle: zero (see Figure 8a) and 20° (see Figure 8b). Both point clouds, obtained from the respective free map, can be seen in Figure 8c, where their fitted linear equations appear as red lines. It is important to highlight that these linear equations have different values of the slope ($C_{\Delta }$) due to the different values of the roll angle (see Equation (18)). However, both linear equations have a closed y-intercept ($d_{\Delta }$), which shows the robustness of the method for the pitch angle self-calibration (see Equation (19)).

## 4. Results and Discussion

### 4.1. Assessment of the Method

**Figure 9:**
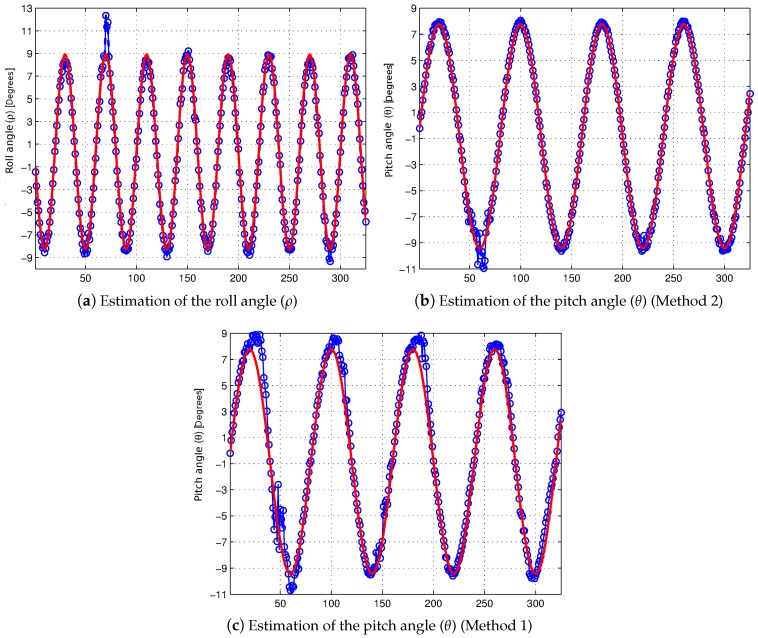
Self-calibration results of the roll angle (*ρ*) and the pitch angle (*θ*). The estimated values appear in blue and the ground truth in red. Results by using Method 2 (**a**,**b**). Results by using Method 1 (**c**).

The synthetic sequence [10] is used again in order to quantify the efficiency of the presented method for the camera pose estimation, when significant variations exist in the value of the roll angle (*ρ*). This synthetic sequence has been modified so as to vary the values of the extrinsic parameters (h,θ,ρ) along the sequence, and as a result, a ground truth is generated to assess the self-calibration method. The value of the roll angle (*ρ*) has been modified along the sequence, and it follows a sine function where the values fall below ±9° (see Figure 9a).

Firstly, we assess what impact the variations in roll angle (*ρ*) have on the estimation of both the pitch angle (*θ*) and the roll angle (*ρ*). Figure 9 shows the results of the self-calibration of the roll angle (*ρ*) (Figure 9a) and the pitch angle (*θ*) (Figure 9b) by using Method 2 (non-negligible values of the roll angle). On the other hand, the result of the self-calibration of the pitch angle (*θ*) is also shown in (Figure 9c) by making use of Method 1 (negligible values of the roll angle) in this case. Regarding the self-calibration of the roll angle (*ρ*), the good result achieved in the estimation should be noted, except for some outliers in the absence of enough points belonging to the road due to a large obstacle ahead (usually vehicles), which occludes the road in the image almost entirely. The error along the sequence is analyzed in order to quantify these good results of the estimation. The average error of the self-calibration of the roll angle (*ρ*) is 0.38°. On the other hand, as might be expected, the self-calibration Method 2 (non-negligible values of the roll angle) has a greater robustness than Method 1 (negligible values of the roll angle) against variations in the value of the roll angle (*ρ*), as can be seen by comparing the results of the self-calibration of the pitch angle (*θ*) by using Method 1 and Method 2 (see Figure 9b,c). From a quantitative point of view, the estimation of the pitch angle (*θ*) by using Method 1 (negligible values of the roll angle) has an average error of 0.69°, whereas the self-calibration method for non-negligible values of the roll angle (Method 2) reduces the average error to 0.20°, i.e., deploying Method 2 implies reducing by two-thirds the average error.

**Figure 10:**
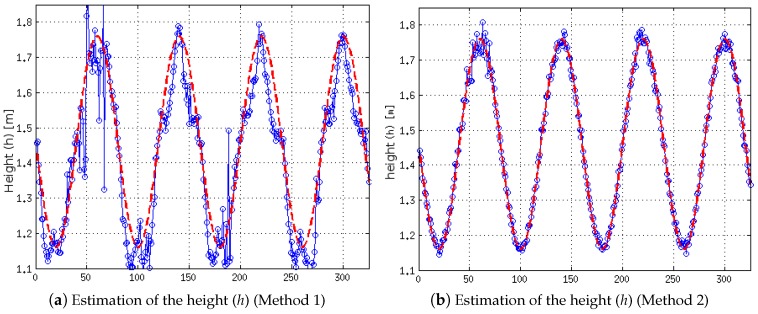
Result of the self-calibration of the height (*h*) for non-negligible values of the roll angle. Estimated values appear in blue and the ground truth in red.

Secondly, we assess the efficiency of the presented method to estimate the value of the height (*h*) where the stereo system is located and what impact the variations in roll angle (*ρ*) have on its estimation. Figure 10 shows a comparison of the results of the self-calibration methods previously described, when the height (*h*) varies between 1.15 and 1.75 m following a sine function in addition to the variation of the roll angle (see Figure 9a). First, the estimation result of Method 1 (negligible values of the roll angle) is shown in Figure 10a), whereas the result of Method 2 (non-negligible values of the roll angle) is shown in (Figure 10b). The best performance of Method 2 can be seen with the naked eye. From a quantitative point of view, Method 1 has an average error of 0.062 m, whereas Method 2 has an average error of 0.012 m, which implies that the average error has been reduced five-fold.

Finally, we assess the impact of the number of road points used for estimation on the efficiency of the presented Method 2 (non-negligible values of the roll angle). The number of road points used in this study varies between 1% and 50% of the available points. Table 1 shows the results of the error of estimating the pitch angle (*θ*) and the roll angle (*ρ*), respectively. The aim of this study is to assess the proper percentage of road points used for the self-calibration method in order to reduce the computational cost of the algorithm in contrast to using all available points (100%) of the road (see the computing time reduction in Table 1). It is possible to reduce the proportion of road points used in order to estimate the extrinsic parameters, and it does not worsen the error substantially, as is shown in the table. The proper percentage of available points of the road to be used so as to estimate the extrinsic parameters has been fixed to 10%. This value has been used to obtain the results presented until now, as well as in the subsequent results.

### 4.2. Comparison with Methods of the State of the Art

The presented method of the estimation of the extrinsic parameters (height (*h*), pitch angle (*θ*) and roll angle (*ρ*)) will be compared in this section with a relevant algorithm [11] belonging to the topic of camera pose estimation. This algorithm makes use of the road geometry, which is assumed flat and ahead of the vehicle, by using the information of the disparity map, i.e., this algorithm is based on the same premises as our self-calibration method, so this is a worthwhile comparison.

**Figure 11:**
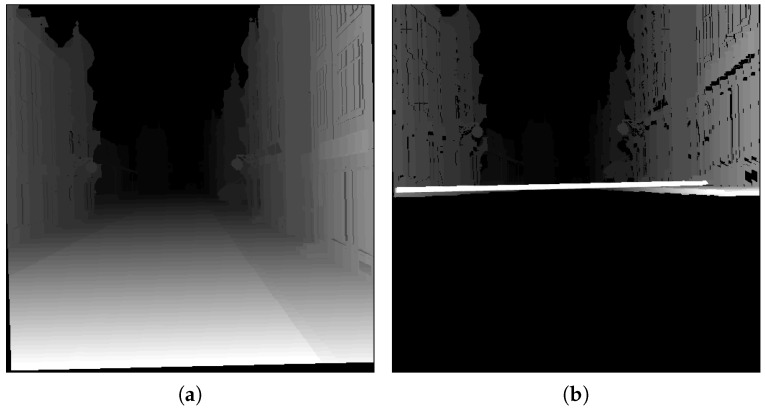
(**a**) Original disparity map (equalized); (**b**) virtual disparity map (equalized).

The algorithm presented in [11] is based on the technique known as virtual disparity map (see Figure 11), which consists of achieving an equivalent disparity map assuming that the stereo system is set at the ground level. The disparity map in each frame (*t*) is transformed into the virtual disparity map by means of the values of the extrinsic parameters estimated on the previous frame (t−1). After obtaining the virtual disparity map, it is used to generate the v-disparity. From the v-disparity, it is possible to estimate the variation of the height (Δh) and the pitch angle (Δθ) between consecutive frames (t−1 and *t*) in a similar way to Method 1. Once the variations in the height and the pitch angle have been obtained, a new projection onto the u–v plane is performed, where the road appears as a straight line (see Figure 11b), and it is possible to estimate the variation of the roll angle (Δρ) between consecutive frames (t−1 and *t*). The chief differences between both methods lie in the fact that Method 2 uses the free map instead of the disparity map and also that it does not need to know the values of the extrinsic parameters of the previous frame (t−1).

The comparison with this algorithm is quantitative, where Table 2 shows the error statistics in the estimation of the extrinsic parameters (ρ,θ,h) by means of this algorithm [11] along the test sequence. In order to facilitate the comparison, the error statistics by using the method presented (Method 2) have been also presented in Table 2. As shown by the data, the presented method achieves more accurate results when it comes to estimating the pitch angle (*θ*) and the height (*h*), whereas the results of the roll angle (*ρ*) estimation are slightly less accurate. In order to assess the effect on the algorithm [11] results of using the free map instead of the disparity map, the results obtained with the synthetic sequence are also presented in Table 2. As can be noted from the use of the free map in the algorithm [11], the results obtained are very similar both for height (*h*) and for pitch angle (*θ*), whereas the estimation of the roll angle (*ρ*) is relatively improved.

**Figure 12:**
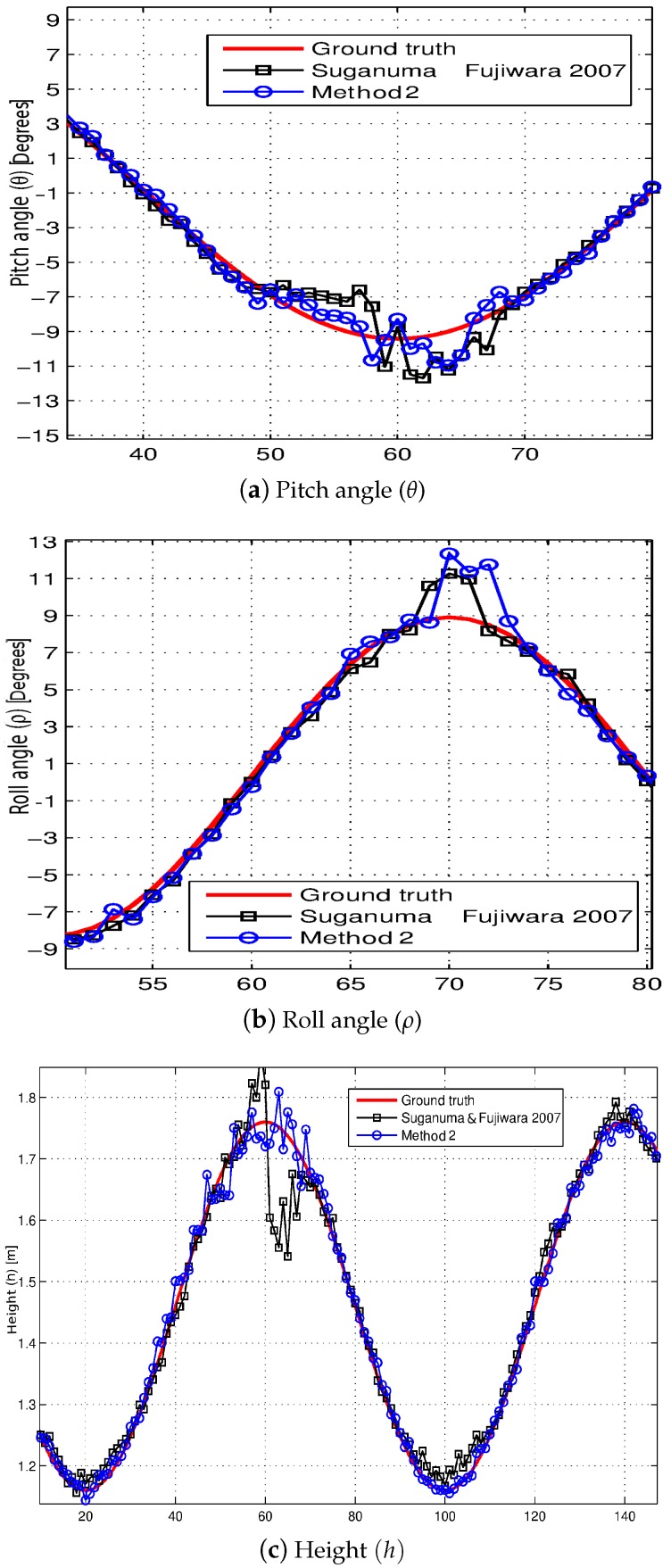
Comparison between the self-calibration results obtained by using the presented Method 2 and the method based on the virtual disparity map presented in [11].

Figure 12 depicts the results of both self-calibration methods on certain points of interest of the sequence. In line with the data of Table 2, it is possible to see a better performance of the presented Method 2 with respect to the method based on virtual disparity map [11], when both the pitch angle (*θ*) and the height (*h*) are estimated (see Figure 12a,c), where the self-calibration of the height (*h*) by using the method presented (Method 2) is closer to the ground truth, whereas the results of the roll angle estimation (*ρ*) are slightly less accurate (see Figure 12b).

### 4.3. Experimental Results

**Figure 13:**
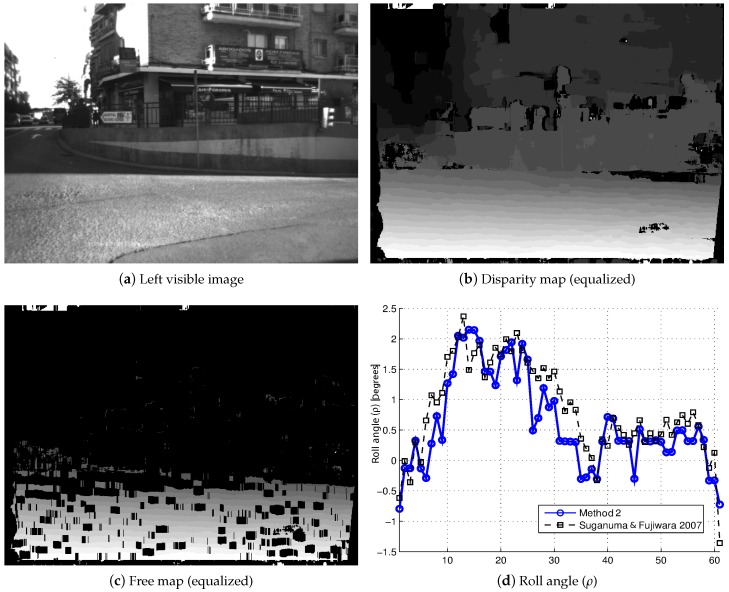

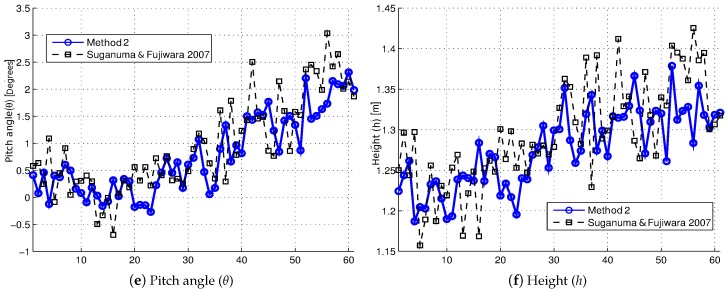
Self-calibration results of the extrinsic parameters (ρ,θ,h) in a real traffic environment (Example 1).

**Figure 14:**
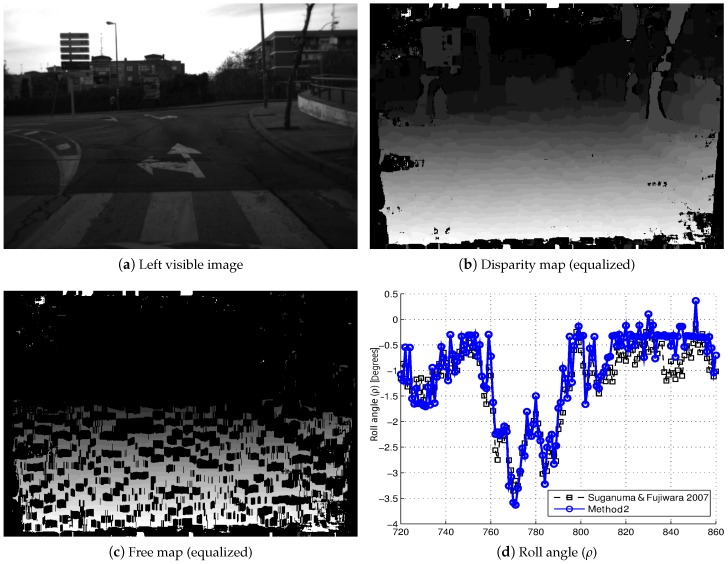

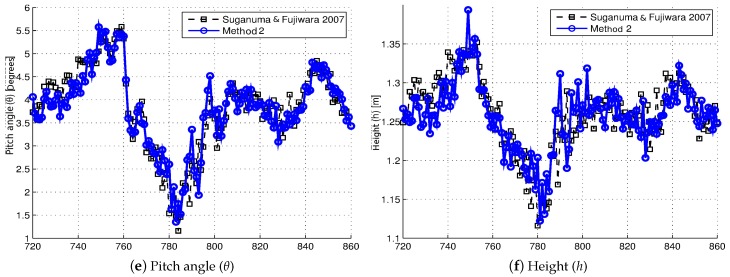
Self-calibration results of the extrinsic parameters (ρ,θ,h) in a real traffic environment (Example 2).

Once the performance of our self-calibration method has been tested, it is applied to real images captured in traffic environments. Figure 13 shows the results achieved by this method along a sequence of 60 stereo pair images, which have been captured in a real traffic environment, where the road has a slope towards the right (see Figure 13a). The vehicle turns to the right along the sequence, in such a way that this initial slope of the roll angle (*ρ*) is translated into a variation of the pitch angle (*θ*) while the vehicle is in motion, as we can see in the self-calibration results of the roll angle (*ρ*) (Figure 13d) and the pitch angle (*θ*) (Figure 13e). Another example is shown in Figure 14: in this case, the sequence consists of 120 stereo pair images, where the vehicle drives through a blind hill, which has a slope towards the left in its top (see Figure 14a). It is possible to see the variations that appear in the extrinsic parameters (pitch angle (*θ*), roll angle (*ρ*) and height (*h*)) in the results of the self-calibration method presented (see Figure 14e–f). For both examples, the disparity maps (Figure 13b and Figure 14b) and free maps (Figure 13c and Figure 14c) are also shown. It is important to highlight that these results have been achieved by using a 10% of the available road points, which reduces the computational cost of the implementation. The results obtained by the self-calibration algorithm [11] (dashed line in black) are also shown for comparison purposes.

## 5. Conclusions

Being able to self-calibrate the pose of the stereo system with respect to the road or ground ahead of the vehicle at all times provides us with critical information, since the degree of accuracy of the measurements of the distance to the environment elements has a huge impact on the decision-making process of ADAS or autonomous vehicles. A lack of accuracy in measuring the distances to the environment elements can have serious consequences for people and vehicles in traffic scenarios.

A new method of camera pose estimation has been presented in this paper, whose main contribution to the state of the art is that high values (±9°) of the roll angle (*ρ*) have no impact on estimating the pitch angle (*θ*) of the stereo system with respect to the road. Firstly, a mathematical formulation has been developed as a starting point in order to obtain the relationship between image coordinates and world coordinates. This new method has been compared with a previous work [9], which is affected by high values of the roll angle (*ρ*). The presented method has shown an improvement in the results: self-calibration error in the pitch angle (*θ*) is reduced by 70%, and the self-calibration error in the height (*h*) is reduced by 80%. In turn, the presented method has been also compared with a relevant self-calibration method in the topic [11]. Regarding the method [11], an improvement in the result rates has been also achieved in the case of estimating the pitch angle (*θ*) and height (*h*) (reduction of self-calibration error: pitch angle (*θ*) 35% and height (*h*) 33%), whereas the self-calibration results of the roll angle (*ρ*) are slightly less accurate (increment of the self-calibration error of the roll angle (*ρ*) by 5%). A synthetic sequence [10] has been used as a tool for comparison, which has been modified following a ground truth so as to change the values of the extrinsic parameters (θ,ρ,h). The comparison has been enriched by the experimental results of the self-calibration method while an intelligent vehicle has been tested in real traffic environments.

Finally, future works will be oriented toward the filtering of the self-calibration results by using UKF (Unscented Kalman Filter) in order to reduce the effect of possible outliers in the estimation. In addition, an assessment will be performed of what impact the self-calibration has on the results of different algorithms applied to autonomous vehicles, such as visual odometry or SLAM (Simultaneous Localization And Mapping).

## Figures and Tables

**Table 1:** Quantitative analysis of the error obtained in the estimation of the pitch (*θ*) and roll (*ρ*) angles for different numbers of road points used.

% Points Used	50%	25%	10%	5%	1%
Pitch angle average error (°)	0.1856	0.1751	0.1985	0.2174	0.2939
Roll angle average error (°)	0.3361	0.3598	0.3791	0.3771	0.3894
Computing time reduction (%)	58.0	72.9	78.16	79.56	80.49

**Table 2:** Statistics error of the self-calibration of the extrinsic parameters by using Method 2 and the algorithm presented in [11] (virtual disparity map).

Method 2	Roll Angle (*ρ*)	Pitch Angle (*θ*)	Height (*h*)
Mean	0.38°	0.20°	0.012 (m)
**Virtual disparity map**	**Roll Angle** (ρ)	**Pitch Angle** (θ)	**Height** (***h***)
Mean	0.36°	0.27°	0.018 (m)
**Virtual free map**	**Roll Angle** (ρ)	**Pitch Angle** (θ)	**Height** (***h***)
Mean	0.33°	0.28°	0.017 (m)
